# Quantitative Measurement of the Pressure and Shear Stress Acting on the Body of a Wheelchair User Using a Wearable Sheet-Type Sensor: A Preliminary Study

**DOI:** 10.3390/ijerph192013579

**Published:** 2022-10-20

**Authors:** Satoshi Shirogane, Shigeru Toyama, Motonori Hoshino, Atsushi Takashima, Toshiaki Tanaka

**Affiliations:** 1 National Rehabilitation Center for Persons with Disabilities, 4-1 Namiki, Tokorozawa 359-8555, Saitama, Japan; 2 Institute of Gerontology, The University of Tokyo, 7-3-1 Hongo, Bunkyo-ku, Tokyo 113-8654, Japan; 3 Department of Physical Therapy, Faculty of Health Sciences, Hokkaido University of Science, 7-Jo 15-4-1 Maeda, Teine, Sapporo 006-8585, Hokkaido, Japan; 4 Graduate School of Biomedical Engineering, Tohoku University, 6-6-12 Aramaki Aza Aoba, Aoba-ku, Sendai 980-8579, Miyagi, Japan

**Keywords:** wearable sensor, shear force, electric wheelchair, pressure ulcer

## Abstract

To provide a safer sitting environment for wheelchair users, it is important to quantitatively measure the forces acting on the contact surface between the seat and the person in the wheelchair. In addition to the pressure acting on the buttocks, shear forces have received particular attention in recent years; however, measuring shear force is more difficult than measuring pressure. To obtain this measurement, a thin and flexible sensor that can be used in a natural state on a wheelchair is needed. Therefore, we constructed a measurement system using our previously developed wearable sheet-type sensor (0.9 mm thick). In this study, preliminary tests were conducted using human dummies before testing on humans. Sensors were placed in four locations on the humanoid dummy’s back and buttocks, and the electric wheelchair was tilted and reclined five times each. The results showed that the sensor output pattern was reproducible and valid enough to proceed to the next step. However, the shear force in the internal and external directions was greater than expected, which indicates that the equipment and testing methods must be reviewed. On the basis of the results obtained in this preliminary study, preparations will be made for testing on human subjects.

## 1. Introduction

For wheelchair users, pressure ulcers on the supporting surface of the buttocks are a serious risk and require special attention [1]. Previous studies have reported a high frequency of pressure ulcers in older adults and in those with spinal cord injuries who are unable to walk without assistance [2,3,4]. Once ulcers develop, they require significant treatment and greatly reduce an individual’s quality of life [5]. Pressure ulcers are considered to develop when external pressure is applied to a part of the body and blood flow to the soft tissues in that area is reduced or stopped. If this condition continues for a long time, the tissue becomes irreversibly damaged from ischemia [6]. Although the exact mechanism of the condition is still not fully understood, pressure and a number of other complex factors can be cited as causative factors. Therefore, seating interventions are commonly used in clinical practice to select and adjust wheelchairs and cushions based on the subject’s physical and living conditions. This method distributes the pressure on the subject’s body surface and prevents pressure from being concentrated in specific areas. In addition to such pressure redistribution, measures have been recommended such as reducing the amount of time the subject is seated in a wheelchair [7], but only limited measures can be selected in the field.

Another measure often used is changing the sitting posture in the wheelchair, such as tilting and reclining. In a wheelchair, reclining means changing the angle between the back support and the seat surface, while maintaining the angle between the seat surface and the ground, and tilting means changing the angle between the seat surface and the ground, while maintaining the angle between the back support and the seat surface. This postural transformation avoids prolonged pressure concentration in specific areas and improves blood flow. Jan et al. [8] found that, to promote skin blood return in patients with spinal cord injury to reduce the risk of pressure ulcer development, the wheelchair tilt space must be at least 35° to promote skin perfusion over the sciatic tuberosity when combined with a 100° recline, and must be at least 25° when combined with a 120° recline. Additionally, Jan et al. [9] reported that, in patients with spinal cord injury, greater angular space tilt and recline are required to improve muscle perfusion, compared with skin perfusion. These postural changes may be manipulated by the electric wheelchair users themselves. However, care must be taken to achieve adequate results.

Otherwise, it has been reported that both pressure and shear forces, which inhibit blood flow, are factors in pressure ulcer development [10]. Furthermore, researchers have reported that when a shear force is high, vascular occlusion occurs at half the pressure of a low shear force, resulting in the viewpoint that this shear force is a more important factor [11]. This suggests that the effect of shear forces on the obstruction of blood flow may be a significant factor in pressure ulcers, and efforts have been made to determine the effect of shear forces acting on the buttocks of wheelchair users [12,13,14,15]. However, one problem in the way of research progress is the difficulty in measuring shear force in wheelchair users, compared with pressure. There is a need for thin, flexible sensors that can be placed between the user and the wheelchair to allow measurements in a more natural state. Therefore, we developed a thin and flexible sensor that can measure shear force [16] and simultaneously measure tri-axis loads with added pressure [17]. Furthermore, we are working on a system that uses these sensors to quantitatively and continuously measure pressure and shear forces on the surface of a wheelchair seat.

In preparation for the human application of the constructed system, we conducted preliminary experiments using a humanoid dummy. The purpose of this study is to visualize continuous changes in pressure and shear forces that act on the wheelchair and body contact surfaces during common wheelchair postural maneuvers (tilting and reclining), and to confirm the following two points. The first is to verify that the pressure and shear force measured between the human dummy and the wheelchair repeatedly exhibit similar values over multiple measurements. The second is to verify that the measured time series data show changes corresponding to the posture transformation of the wheelchair. Since tilting and reclining are two typical postural maneuvers for wheelchairs, these two patterns were first targeted for this preliminary test.

In the future, we anticipate that the seat angle will be automatically controlled based on these measured values; however, this research is still in its early stages. We previously measured the shear force on the buttocks when a healthy subject tilted the upper body voluntarily [15]. However, this preliminary study simulates the body being moved by others, without the subject moving themselves.

## 2. Materials and Methods

In this preliminary test, we attached the developed sensor system to an electric wheelchair. We placed a humanoid dummy on the sensor to measure and visualize the contact pressure and shear force data while tilting and reclining the seat.

### 2.1. Humanoid Dummy

The dummy used was a humanoid dummy with a built-in skeletal model (Humanoid Dummy β Full Skeleton, HBD-0800-00, Avis Corp., Tokyo, Japan). The dummy is purported to have human-like flexibility and a range of joint motion, approximating that of a human body, covering approximately 95% of the moving parts of the average human body. Most joints are movable in much the same way as in the human body. The scapula is interlocked when the upper arm is raised, but the temporomandibular and sacroiliac joints are not movable. The body surface is covered with a soft urethane sponge, and almost all of the landmark points of the human body can be confirmed by touch. When pressed with the hand, the dummy’s body surface concaves, like that of a real human, and returns to its original shape when the pressing hand is released. The size of the dummy was designed to mimic a male of approximately 160 cm in height and 55 kg in weight, and the actual weight was approximately 12 kg.

### 2.2. Sensor and Measurement Equipment

**Figure 1:**
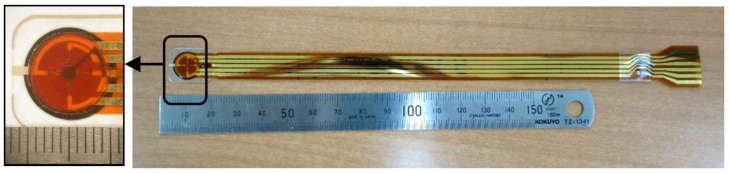
Tri-axis load sensor. Overview of the tri-axis load sensor.

**Figure 2:**
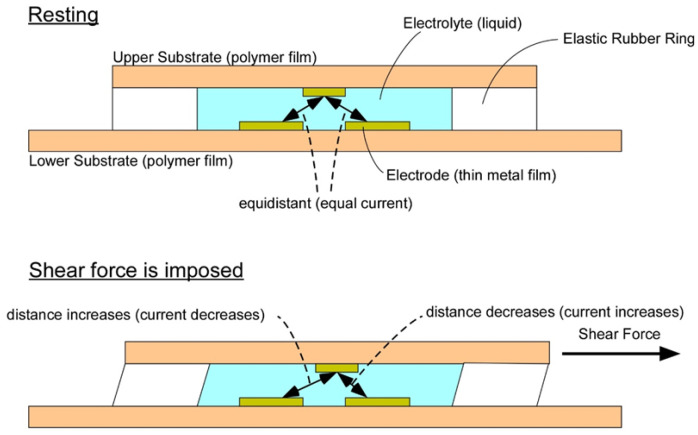
Schematic diagram of the shear force sensor cross-section, demonstrating the working principle of the sensor.

The sensor used in this study was a thin, flexible sheet-type sensor that was a hybrid of two types of sensors: a commercially available sheet-type pressure sensor (FlexiForce, A201/4.4 N, Tekscan Inc., Norwood, MA, USA) and a previously developed shear force sensor (Figure 1) [17]. Various thin sensors for pressure measurement are already available on the market, but thin sensors for shear force measurement are generally difficult to obtain, so a hybrid-type thin sensor was used. The sensor, which has a diameter of 10 mm, is depicted in Figure 1. With a thickness of 0.9 mm for the measurement portion of the sensor, the sensor was capable of simultaneous tri-axial measurement of pressure and shear in two axial directions. The measurement of shear force is based on our original principle [16]. The operating principle of the sensor is depicted in Figure 2. The shear force sensor is made up of two sheet-type electrode substrates that face each other and are separated by a rubber ring. An electrolyte is injected into a disk-shaped space between the rubber ring and the electrode substrate. Therefore, when a voltage is applied between the electrodes on the upper and lower substrates, current flows. When a shear force, i.e., a force parallel to the substrate, is applied, the distance between the upper and lower electrodes changes, as does the current. As a result, the shear force can be calculated by measuring the current. Shear force is a two-dimensional force, but as illustrated by the photograph in Figure 1, four electrodes are formed on one side and one electrode is formed on the other side, so the currents between the two components can be measured simultaneously. According to Reference [16], the relationship between shear force applied to the sensor and sensor response is experimentally linear, up to a force of 5 N.

We also developed a multi-sensor measurement system, which consisted of a sensor-driven circuit and computer software. The data sampling rate was set to 5 Hz. The sensors were connected to a PC via Bluetooth technology, and the transmitted signals were digitized as a value (N), representing the directional force applied to each axis, using proprietary software. The sensor was placed at four key pressure ulcer prevention points: Ch1, the subscapular angle; Ch2, the lumbar back; Ch3, the ischial tuberosity; and Ch4, the thigh. 

The left side is an enlarged photograph of the main part of the sensor. As shown in this enlarged photograph, the shear force sensor is laid on top of the pressure sensor. The signal lines of the shear force sensor and those of the pressure sensor are put together at the terminal of the right side. The terminal is connected to a normal signal cable (ca. 1 m) via a connector, and the cable is connected to a circuit box (installed on the back of the electric wheelchair).

**Figure 3:**
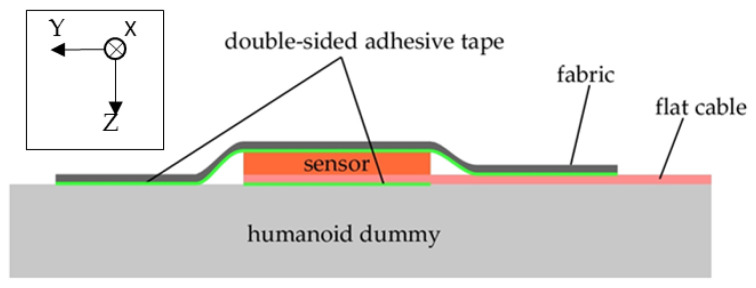
Method to attach the sensor to the human dummy. Note: A diagram showing how to attach the sensor to the humanoid dummy from a cross-sectional view.

The sensor was fixed to the body surface of the dummy using double-sided tape, and fabric (material: nylon/polyurethane, cut from a commercial stocking, and approximately 0.15 mm thick) was stretched over it to prevent it from catching on the wheelchair’s support surface during measurement. The sensor’s surface and the stockings were attached to each other using double-sided tape (Figure 3). The sensor was integrated with a flat cable (~20 cm in length), and the other end of the flat cable was connected to the measurement circuit box via a normal cable. The circuit box was fixed to the rear of the wheelchair, and the cable was taped to the wheelchair so that it would not get in the way when changing the posture of the humanoid dummy. The circuit box was powered by a mobile battery and was wirelessly connected to a personal computer, as described above. Therefore, the entire experimental system was physically independent of other measurement systems and was easy to handle during experiments. 

### 2.3. Electric Wheelchairs and Posture Transformation

**Figure 4:**
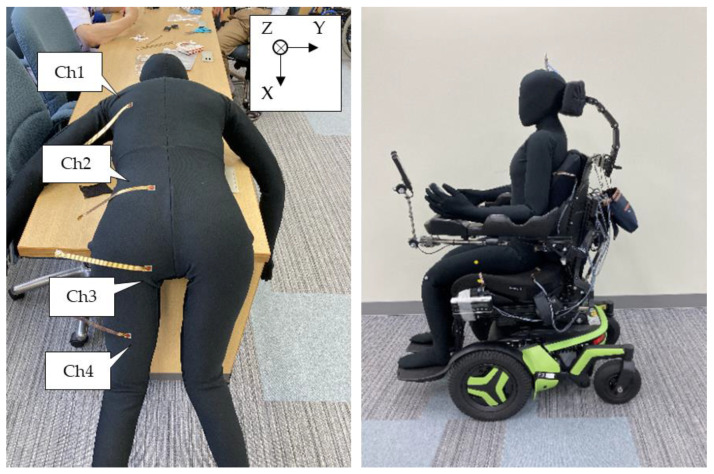
Experimental setting. (**Left**) dummy with the sensor attached. Note: to make it easier to understand the position of the sensor, the state before fabric attachment is shown. (**Right**) electric wheelchair with the dummy. Multiple markers are affixed to the sides of the dummy and wheelchair to monitor posture.

We used an electric wheelchair (F3 Corpus, Permobil Inc., Timrå, Sweden) with a cushion (Corpus Ergo Air Cushion, Permobil Inc., Timrå, Sweden) on its seat and a humanoid dummy (Figure 4). 

**Figure 5:**
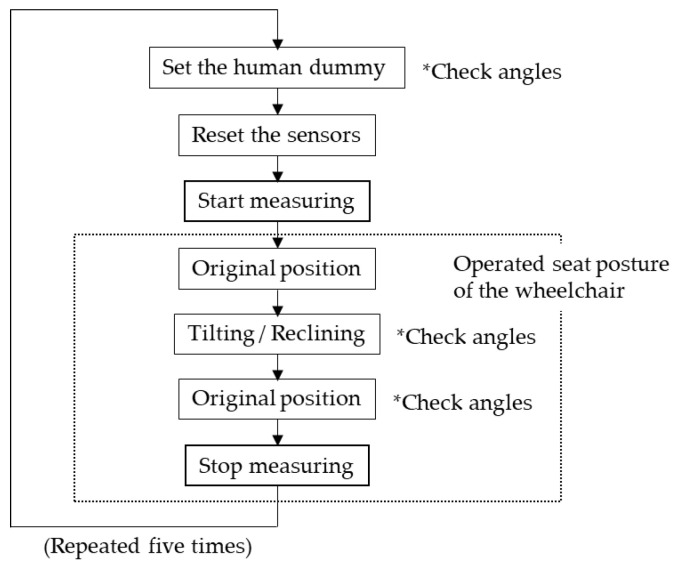
Explanation of the measurement.

The examiner manually operated the angle of the back support and seat using the controller of the electric wheelchair, while also checking the change in that angle with a three-dimensional position-measuring device (V120: Trio, OptiTrac Inc., Corvallis, OR, USA) using light-reflective markers installed on the wheelchair. Five measurements were taken for each of the two patterns, in which the backrest was tilted backward 60° from its initial position (approximately 90° for both back and seat) and returned to its original position, and for the tilting pattern, in which the seat was tilted backward 30° from its initial position and returned to its original position. The dummy was lifted once on each trial and then returned to a sitting position to release the force remaining on the contact surface. The sensor was zero-corrected at the point of sitting up, and this was used as the initial value from which changes were measured (Figure 5).

### 2.4. Data Analysis and Visualization

We stored data collected from the sensors in the comma-separated value format. The force data of the tri-axes were defined as “x-axis” for the longitudinal direction of shear force “y-axis”, for the mediolateral direction, and “z-axis” for pressure. These directions were defined with respect to the forces applied to the sensor. To confirm the approximate measurement validity, the data were overlaid as a time series graph for all five trials for each axis direction. In the statistical analysis, we extracted data that showed well-represented changes in values due to posture, aligned the baselines, and calculated the standard deviation of data from five measurements of the typical part to confirm that the values were within 20% of the measurement’s minimum–maximum range.

Additionally, for a more comprehensive view of the magnitude of the forces and their changes in the three axial directions for a typical trial, the data were visualized as a time series graph, with the vertical axis as pressure and the two shear force axes as vectors starting from the plot points.

## 3. Results

All data were successfully recorded without omissions in a total of 10 trials—5 for each of the two patterns of postural transformation.

### 3.1. Measurement of Forces in Each Axial Direction and Repeatability

**Figure 6:**
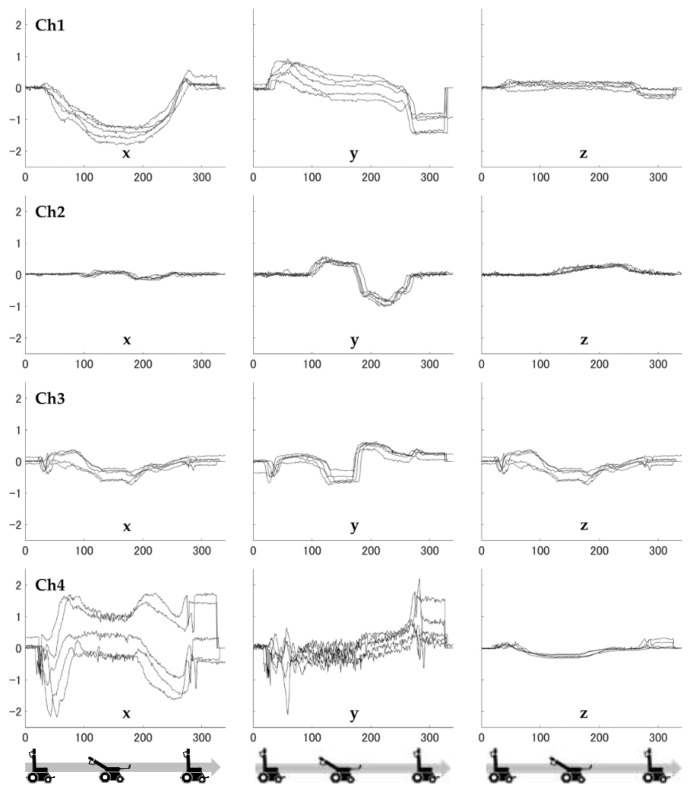
Load values for the three axes at each sensor (reclining pattern).

**Figure 7:**
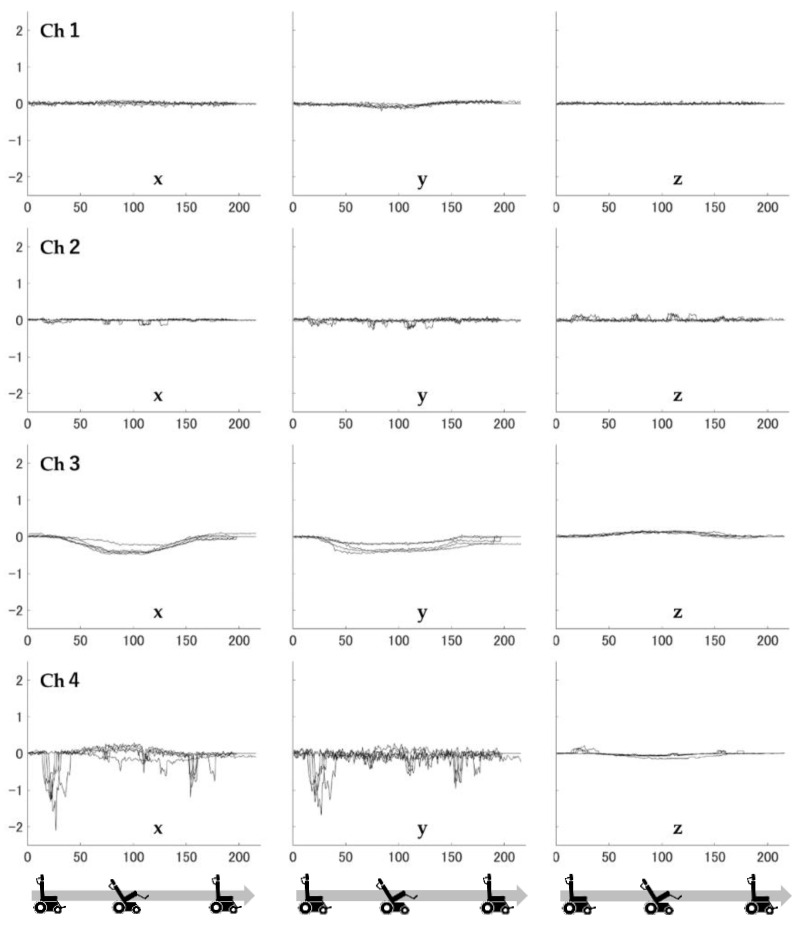
Load values for the three axes at each sensor (tilting pattern).

The time variation of the measured values for each axis is shown in Figure 6 and Figure 7. The former is a reclining pattern, and the latter is a tilting pattern. In Figure 6 and Figure 7, the horizontal axis shows time and the vertical axis shows force. The direction of force is positive for the foot side and negative for the head side in the longitudinal direction, and positive for the inside and negative for the outside in the mediolateral direction. In Figure 6, for each of the five repeated trials, a nearly identical pattern was observed for each force trajectory. In Figure 7, as with the reclining pattern, these forces showed a similar trajectory for the five trials, and the force values recorded when tilting were generally smaller than those when reclining, with the exception of sensor Ch4. Furthermore, the standard deviations of 20 data points in the x-axis (shear force) were 0.08 N, 0.04 N, 0.05 N, and 0.39 N for before and after the point at which the back support was fully reclined and for the reclining pattern, where there were large changes in measured values, respectively. The percentages of the maximum and minimum ranges were 4.9%, 18.5%, 7.6%, and 27.8%, respectively.

### 3.2. Direction of Force and Its Change in a Typical Example

**Figure 8:**
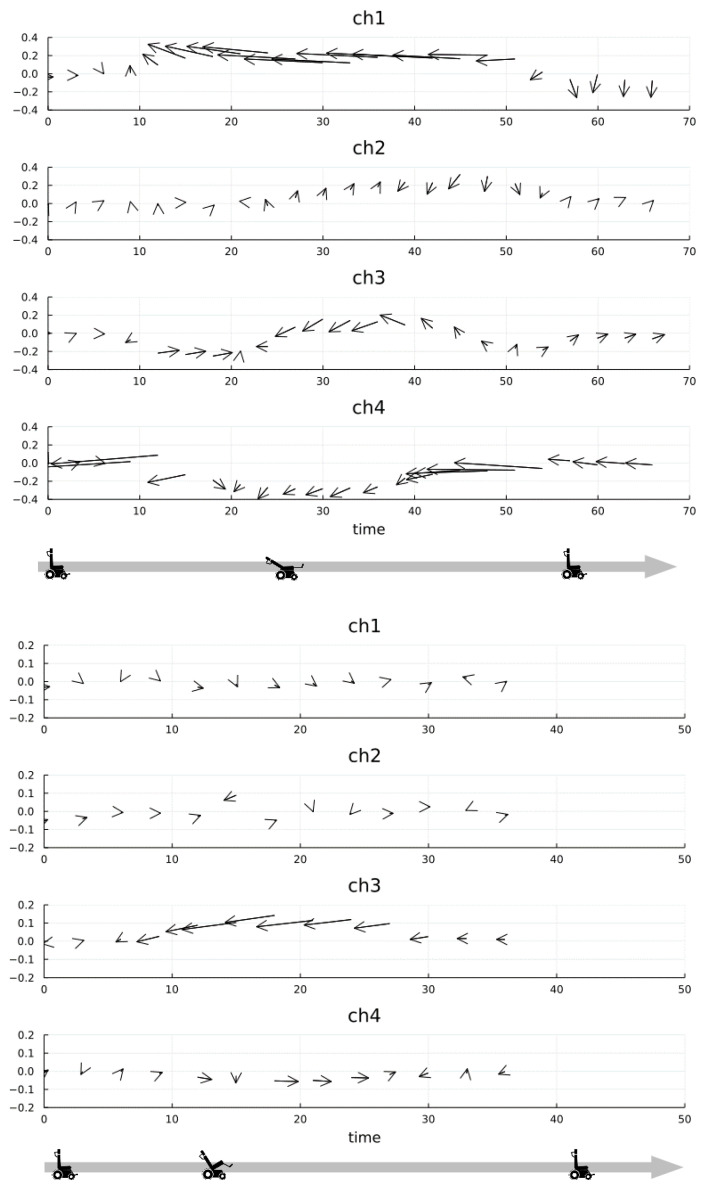
Vector representation of forces in tri-axes (**upper** graphs—reclining; **lower** graphs—tilting).

Figure 8 shows a vector representation of the combined tri-axial forces with respect to a typical one from the five-trial data. In Figure 8, the horizontal axis shows time and the vertical axis shows force. The arrows represent a two-dimensional vector of shear forces, with the pressure value (z-axis) as the starting point. The length of the arrow represents the magnitude of the shear force vector, and the direction of the arrow represents the direction of the shear force vector. The horizontal component of the arrow corresponds to the x direction in Figure 6 and Figure 7 (positive—foot side; negative—head side), and the vertical component corresponds to the y direction in Figure 6 and Figure 7 (positive—medial side; negative—lateral side). The length scale of the vector is the same as the pressure (z-axis). In the upper part of Figure 8, regarding the recline pattern, a large shearing force was observed in the longitudinal direction on Ch1 and Ch4. In the lower part of Figure 8, regarding the tilting pattern, all forces showed smaller values to those in the recline pattern; however, in Ch3, we observed that only the shear force in the longitudinal direction increased and then decreased.

## 4. Discussion

In this study, we confirmed that the sensor system we constructed was capable of continuously collecting quantitative data during postural changes in a wheelchair. The recorded data showed similar trends over multiple trials and seemed to be reproducible. The x-axis data for Ch4 in the reclining pattern showed more variation in measured values than the others. However, when the baselines were aligned, the overall trend was similar and appeared to be influenced by the variation in the initial values. One of the limitations of this study is that variations in the mechanical initial conditions of the human dummy may have influenced the measurements, which could not be fully unified.

The fact that relatively large forces were observed on Ch1 and Ch4 during reclining was considered a reasonable result, because the shearing force increased because of the deviation between the position of the center of rotation of the human body and the wheelchair. Additionally, the force was smaller when tilting than when reclining, and only the displacement of the ischial nodule appeared large (Figure 8, Ch3). This was considered appropriate in terms of the tilting characteristics, as the body does not move on the seat and the load site changes only when the direction of gravity changes.

However, it should be noted that the test we conducted used a humanoid dummy, which is fundamentally different from an actual person. First, the dummy used in this study weighed approximately 12 kg, which is approximately one-fifth of the weight of the human model. Although the skeletal model is built into the dummy and mimics bone protrusion, joint motion, and so forth, the lumbar vertebrae are not mobile, and the soft tissues are composed of urethane sponge, which have different characteristics from those of a real person. In particular, the fact that the dummy is considerably lighter than a human is expected to have a significant effect on changes in pressure and shear force.

We also found some concerns regarding points other than the dummies. We repeatedly observed spike-like data over a relatively long period in multiple trials, so there may not be an instantaneous displacement (i.e., transition point from static to dynamic friction). Additionally, although we zero-corrected the sensor before each measurement, it may have been more appropriate to zero-correct the sensor with no load, to confirm the true magnitude and direction of the force in this case. Furthermore, the fact that the y-axis forces (in the internal and external directions) were unexpectedly large suggests that the experiment may not have been set up well enough. To set up the dummy in a sufficiently neutral condition, the examiner may need to not only confirm this by observation, but also devise a more appropriate initial condition.

The preliminary findings indicate that the measurement system, based on the wearable seat-type sensor developed by us, has reasonable repeatability and that the initial state of the subject’s sitting affects the subsequent measurement results. The latter problem can arise when applied to actual users, and clarification of this point was especially clinically significant. Furthermore, the equipment and testing methods will be reviewed, and preparations will be made for human subject testing. In the future, the development of such measurement devices is expected to progress further and contribute to the safety of wheelchair users.

## 5. Conclusions

In this preliminary test using humanoid dummies, our system with wearable sheet-type sensors showed similar trends in repeated trials. This might indicate that the system is valid for measuring the pressure and shear forces acting on the contact surface between the person sitting in the wheelchair and the seat surface. This will assist in more effective planning of human trials and will help improve the safety of wheelchair users in the future.
